# The effect of *Channa striata*, *Moringa oleifera*, and *Curcuma xanthorrhiza* extract on accelerating recovery in a ventilated patient with hemorrhagic shock grade 3 due to prolonged retained placenta: a case report

**DOI:** 10.1186/s13256-024-04360-2

**Published:** 2024-03-19

**Authors:** Theresia Monica Rahardjo, Aloysius Suryawan, Nadya Rosa Amelia Rumouw, Hizkia Santoso, Amadea Jovita Miko Faustin

**Affiliations:** Faculty of Medicine, Maranatha Christian University, Suria Sumantri 65, Bandung, West Java 40164 Indonesia

**Keywords:** Hemorrhagic shock, *Channa striata*, *Moringa oleifera*, *Curcuma xanthorrhiza*, Antioxidant, Case report

## Abstract

**Background:**

Most of critically ventilated patients with severe hemorrhagic shock experience metabolic acidosis, hypoalbuminemia, electrolyte imbalance, and increased production of free radical. *Channa striata* has a high content of albumin, an essential binding protein that contributes to homeostasis, and when combined with *Moringa oleifera* and *Curcuma xanthorrhiza*, they act as powerful antioxidants. Administration of *C. striata*, *M. oleifera*, and *C. xanthorrhiza* extract orally may benefit patient with hemodynamic issues, including significant blood loss.

**Case report:**

A 40-year-old Indonesian woman came to emergency department with decreased consciousness resulting from hemorrhagic shock grade 3 due to prolonged placenta retention for 10 days after delivery of her third child. She had an emergency hysterectomy and was sent to the intensive care unit with a hemoglobin level of 4.2 gr/dL, despite already receiving two bags of packed red blood cells during operation, and she continued with four more bags within her first day in the intensive care unit. The patient was ventilated, was supported by vasopressors, and had a low albumin level of 2.1 gr/dL. Her hemodynamic profile was difficult to stabilize, with persistent gastric residue and periodic urine output less than 1 cc/kg/hour, thereby slowing the ventilator and vasopressor weaning process. Oral supplementation of *C. striata*, *M. oleifera*, and *C. xanthorrhiza* was given in the second day divided in three doses every 6 hours. After the second dose, gastric residue started to subside and disappeared after the third dose. The patient’s condition improved in the next 24 hours; she was extubated and discharged from the hospital in the fourth day.

**Conclusion:**

This is the first case report describing the effect of *C. striata*, *M. oleifera*, and *C. xanthorrhiza* extract in a patient with severe hemorrhagic shock due to a prolonged placenta. Accelerated recovery showed the possibility benefit of *C. striata*, *M. oleifera*, and *C. xanthorrhiza* extract in stabilizing oncotic pressure, neutralizing free radicals, and preventing further damage in hypoxic cells.

## Background

Hemorrhagic shock due to placenta retention is a common cause of obstetrical morbidity and mortality. Approximately 1–3% of deliveries are affected by a retained placenta, and it is the second leading cause of postpartum hemorrhage (PPH). Prolonged placenta retention can lead to continuous bleeding and massive blood loss, resulting in hemorrhagic shock, which is characterized by a decrease in tissue perfusion, hemodynamic instability, unconsciousness, and death. A study found women with a retained placenta significantly required more blood transfusions (13% in the case group versus 0% in the controls) than with normal delivery. Other studies estimated blood loss exceeding 500 mL, 1000 mL, and 2000 mL in retained placenta, as high as 33.07 (95% CI 20.57–53.16), 43.44 (95% CI 26.57–71.02), and 111.24 (95% CI 27.26–454.00) [1, 2].

Critically ventilated patients with hemorrhagic shock may experience metabolic acidosis, hypoalbuminemia, electrolyte imbalance, and high free radical production. Tissue hypoperfusion will suppress aerobic metabolism, in which adenosine triphosphate (ATP) production is disrupted and an excess quantity of radical oxygen species (ROS) is produced. Anaerobic metabolism will activate lactate production and cause metabolic acidosis [1, 2].

Hypoalbuminemia is a risk factor for morbidity and mortality. Albumin is a major carrier protein and stabilizes oncotic pressure, but it is also a potent antioxidant. Albumin is one of the first proteins to be affected by oxidative stress, therefore its redox status is widely used as a biomarker of various pathological conditions. It is known that the percentage of cysteinylated albumin (Cys34-S-S-Cys) is markedly increased in chronic liver and kidney diseases, as well as in diabetes mellitus. It has also been shown that oxidized albumin can be a biomarker of the severity of such diseases, such as hyperparathyroidism, acute ischemic stroke, Parkinson’s disease, Alzheimer’s disease, Duchenne muscular dystrophy, and many other pathological conditions. *Channa striata* contains large number of albumin and can supply the increasing albumin needs of critical patients in the intensive care unit (ICU) [2, 3].

The free radical level is increased in critical patients. *Moringa oleifera* leaf extract is well known for its antioxidant property. It contains ascorbic acid, flavonoids, phenolics, and carotenoid. These substances can neutralize ROS and inhibit the production of lipopolysaccharide (LPS) and proinflammatory mediators expression. *Curcuma xanthorrhiza* also has antioxidant activity through antioxidant curcuminoid, a group of phenolics compounds of curcumin, demethoxycurcumin, and bisdemethoxycurcumin. It can prevent lipid peroxidation and could serve as hepatoprotector [4, 5]. This study is the first case report describing the effect of *C. striata*, *M. oleifera*, and *C. xanthorrhiza* extract in patient with severe hemorrhagic shock due to a prolonged placenta. The improvement of patient condition showed the possible benefit of *C. striata*, *M. oleifera*, and *C. xanthorrhiza* extract in stabilizing oncotic pressure, neutralizing free radicals, and preventing further damage in hypoxic cells, thus accelerating the recovery process.

## Case presentation

A 40-year-old Indonesian woman came to the emergency department with decreased consciousness resulting from grade 3 hemorrhagic shock due to prolonged placenta retention for 10 days after delivering her third child. Her blood pressure was 80/50 mmHg, heart rate was 135 beats/minute, respiratory rate 25–30 breaths/minute, and she had prolonged capillary refill. She has no history of abortion, hypertension, or diabetes mellitus. She had prenatal care twice during pregnancy and delivered her three children by midwife. She underwent an emergency hysterectomy and was sent to the ICU because of massive blood loss, with a hemoglobin (Hb) level of 4.2 gr/dL despite receiving two bags of packed red blood cells (PRC) during the operation and four more bags within her first day in the ICU. The patient was ventilated, supported by vasopressors, treated with broad spectrum antibiotics, fluid resuscitation, and blood transfusions, accompanied by routine ventilator bundle.

The most prominent abnormal laboratory result, besides severe anemia, was a hypoalbuminemia of 2.1 gr/dL followed by an electrolyte imbalance, including hyponatremia 132 mEq/L, hypocalcemia 7 mg/L, and hypomagnesemia 1.6 mg/dL. Despite her vital sign improvement and electrolyte correction treatment, her hemodynamics were difficult to stabilize, with persistent gastric residue and periodic urine output less of than 1 cc/kg/hour during the first 24 hours, which slowed the ventilator weaning process, vasopressor reduction, and gut feeding.

On the second day, the patient was given an oral supplementation containing extract of 7000 mg *C. striata*, 250 mg *M. oleifera*, and 250 mg *C. xanthorrhiza*, divided into three doses every 5 hours. After the second dose, the gastric residue started to subside, and it disappeared after the third dose. Ventilator weaning was accelerated after the second dose, with improvements in respiratory rate, pressure support, and oxygen supply. Blood pressure became more stable, and urine output increased higher than one cc/kg/hour after the third dose, so vasopressors could be decreased.

The patient’s condition improved on the third day. She received three doses of supplements before extubation and six doses of supplements after extubation. Laboratory examination showed improvement in blood parameters, with Hb reaching 9.8 gr/dL and albumin level reaching 2.5 gr/dL, and normal lactate level in the blood gas analysis result. The patient was discharged from the hospital on the fourth day. The oral supplementation of *C. striata*, *M. oleifera*, and *C. xanthorrhiza* appeared to be safe and effective in improving the clinical condition of the patient with hemorrhagic shock due to prolonged placenta retention.

## Discussion

Retained placenta is a serious obstetrical condition that can lead to significant morbidity and mortality. It is the second leading cause of significant, and even fatal, hemorrhage in the obstetric population. A study estimating blood loss exceeding 500 mL, 1000 mL, and 2000 mL in retained placenta, respectively, is as high as 33.07 (95% CI 20.57–53.16), 43.44 (95% CI 26.57–71.02), and 111.24 (95% CI 27.26–454.00). Another study found that women with retained placenta significantly required more blood transfusions (13% in the case group versus 0% in the controls) than with normal delivery. Large cohort studies have confirmed this elevated risk [1].

The situation becomes worse in prolonged retained placenta, as seen in this patient. She had been bleeding for 10 days, aggravated by inadequate feeding since the last trimester of her pregnancy due to lack of knowledge and economic problems. She was immediately rushed to the operating room to undergo a hysterectomy and received two bags of PRC. There is a possibility the Hb level was less than 4 gr/dL before the operation, because after the operation, her Hb level was 4.2 gr/dL. She received four bags of PRC in the ICU and her Hb level reached 10.0 gr/dL after a total of six bags of PRC transfusion. The long period of hypoxia in this patient would have shifted her metabolism to anaerobic metabolism, resulting in increased lactate, metabolic acidosis, and lower adenosine triphosphate (ATP) production.

The patient also had a low albumin level of 2.1 gr/dL. Hypoalbuminemia is a risk factor for morbidity and mortality. Albumin is a major carrier protein that stabilizes oncotic pressure and is also a potent antioxidant. It is one of the first proteins to be affected by oxidative stress, and its redox status is widely used as a biomarker of various pathological conditions. A study found a relationship between the high oxidative process in critically ill patients in the ICU, the role of albumin as an antioxidant, and the antibiotic vasostatin. The high level of oxidative process in patients in the ICU can oxidize vasostatin and eliminate its antibacterial function. This study showed that continuous infusion of 4% albumin reduced the risk of nosocomial infections in patients in the ICU. By mixing albumin with oxidized vasostatin-1 and using a high-performance liquid chromatography (HPLC) method, it was demonstrated that albumin reduced the oxidized form of vasostatin, thereby restoring and increasing its antibacterial properties [2, 3, 6].

Excessive free radical and low albumin level can aggravate the patient’s condition and halt recovery and the ventilator weaning process although adequate and hemodynamic resuscitation is successful, as can be seen in this patient. Common complications, that include acute respiratory distress syndrome (ARDS), acute renal failure (ARF), myocardial depression, and liver dysfunction, can occur in such a patient. This condition associated with the oxidative stress elicited in cells following ischemia or hypoxia and resuscitation known as reperfusion injury [6].

The oxidative stress following reperfusion injury has been linked to a variety of different sources of ROS. Nonenzymatic sources of ROS, such as hemoglobin and myoglobin, which can be released into extracellular fluid after trauma, have a role as potential mediators of reperfusion-induced oxidative stress. The accelerated ROS production in postischemic tissues attributes to one or more enzymes that are capable of reducing molecular oxygen to form superoxide and/or hydrogen peroxide, with the subsequent release of ROS into the intracellular and/or extracellular compartments. The enzyme systems most commonly invoked to explain the accelerated ROS production in postischemic tissues are xanthine oxidase, NADPH oxidase, the mitochondrial electron transport chain, and uncoupled nitric oxide synthase [6, 7].

Based on this condition, this patient required large amounts of antioxidants to neutralize the high level of free radicals in her body, so the oral administration of *C. striata*, *M. oleifera*, and *C. xanthorrhiza* extract in high concentration had the right basis, besides all major treatments (including emergency hysterectomy, blood transfusion, and accurate ICU therapy) that have been performed on this patient. The liquid supplement has a liposomal form for protection from gastric acidity and will be released in the small intestine to increase its availability. Patient clinical improvement characterized by hemodynamic stability, accelerated ventilator weaning, and vasopressor tapering off, accompanied by significant improvement clinical and laboratory parameters (including Hb, blood gas analysis, and reduced Sequential Organ Failure Assessment (SOFA) score), showed the effectivity of albumin in *C. striata* as a major potent antioxidant, along with *M. oleifera* and *C. xanthorrhiza*, in accelerating patient recovery. (Table 1, 2, 3).

**Table 1:** Laboratory parameters of the patient from 27 August 2023 to 30 August 2023

Period	Before oral supplement					3 hours before supplement	After oral supplement			
Laboratory parameter	27 August 2023 (02:00:41 am)	27 August 2023 (03:40:47 am)	27 August 2023 (06:10:48 am)	27 August 2023 (06:46:38 am)	27 August 2023 (09:10:00 am)	28 August 2023 (06:35:00 am)	29 August 2023 (06:20:00 am)	29 August 2023 (11:40:00 am)	29 August 2023 (06:50:00 pm)	30 August 2023 (06:30:00 am)
Hb (g/dL)	4.2		6.9		8.6	10.0	9.7			9.6
Albumin				2.1			2.3		2.5	
pH	7.288	7.417	7.343			7.481	7.498	7.509		7.499
pCO_2_ (mmHg)	40.8	31.4	36.5			32.1	35.3	34.6		34.5
pO_2_ (mmHg)	142	329	236			155	155	224		121
BE (mmol/L)	−7	−4	−6			0	4	5		4
HCO_3_ (mmol/L)	19.8	20.3	20.0			24.3	27.6	27.8		27.1
tCO_2_ (mmol/L)	21	21	21			25	29	29		28
SaO_2_ (%)	99	100	100			100	100	100		100
Lactate	1.61	1.15	1.21			1.44	1.24	0.64		2.04
SOFA score	10	8	8			7	6	6		1

*BE* Base Excess, *SOFA* Sequential Organ Failure Assessment

**Table 2:** Residue changes before and after oral administration of *Channa striata*, *Moringa oleifera*, and *Curcuma xanthorrhiza*

Period	Before oral supplement				Supplement first dose	3 hours after first dose (50 cc)	3 hours after second dose (50 cc)	3 hours after third dose (50 cc)
Residue parameter	27 August 2023 (05:42:00 am)	27 August 2023 (01:03:00 pm)	27 August 2023 (01:55:00 pm)	27 August 2023 (06:00:00 pm)	28 August 2023 (08:59:00 am)	28 August 2023 (11:58:00 am)	28 August 2023 (06:00:00 pm)	29 August 2023 (00:00:00 am)
Color	Dark green	Dark brown green	Brown green	Brown green	Light Brown	Brown	Clear	No residue
Quantity	20 cc	5 cc	10 cc	30 cc	40 cc	20 cc	10 cc	0 cc
Photo								
Bowel sound	7×/minute	7×/minute	8×/minute	7×/minute	12×/minute	12×/minute	12×/minute	12×/minute

**Table 3:** Patient’s condition before and after oral administration of *Channa striata*, *Moringa oleifera*, and *Curcuma xanthorrhiza*

Period	Before oral supplement		After oral supplement					
Parameter	27 August 2023 (01:40:00 am)	27 August 2023 (06:00:00 am)	28 August 2023 (09:00:00 am)	28 August 2023 (12:00:00 pm)	29 August 2023 (09:00:00 am)	29 August 2023 (12:05:00 pm)	30 August 2023 (00:00:00 am)	30 August 2023 (07:00:00 am)
Ventilator	P-CMV	SIMV-PS	SIMV-PS	SIMV-PS	Spontaneous	T-piece 8 L/minute	NC 6 L/minute	NC 2 L/minute
Norepinephrine	+	+	+↓	+↓	+↓	+↓	–	–
Blood pressure (mmHg)	126/86	105/75	103/72	101/75	108/77	107/74	110/61	121/72
Heart rate (x/minute)	120	125	70	88	78	77	75	78
Saturation (%)	100%	100%	100%	100%	100%	100%	100%	100%
Urine output (cc/kgBW/hour)	< 1	< 1	≥ 1	> 1	> 1	> 1	> 1	> 1

*P-CMV* Pressure-Continuous Mandatory Ventilation, *SIMV-PS* Synchronized Intermittent Mandatory Ventilation-Pressure Support, *NC* Nasal Cannula, *kgBW* kilogram body weight

*Channa striata* is well known for having a high albumin content. Albumin is a universal molecule in a certain sense, which can bind almost all known endogenous compounds, metal ions, and xenobiotics and possesses a number of enzymatic activities including (pseudo)esterase, paraoxonase, phosphotriesterase, thioesterase, glutathione peroxidase, cysteine peroxidase, and some others. Albumin plays an important role in the antioxidant defense of the body. Albumin has a number of amino acids and amino acid sequences that determine its role in redox processes. It is a sacrificial antioxidant, which takes the brunt of the extracellular component of oxidative stress. Its binding with iron and copper cations reduces their activity heavily and sacrifices itself to prevent reaction between iron and copper with hydrogen peroxide to form toxic hydroxyl radicals (Fenton reaction) [2, 3].

Albumin also acts like a ROS trap mainly due to the free thiol detoxification reaction by formation of thiocyanate, catalyzed in the IIIA subdomain, without group of Cys34 residue, despite other six methionine residues. In physiological conditions, about 80% of all detected plasma thiols are a detoxification reaction by formation of thiocyanate, catalyzed in the IIIA subdomain, but without the albumin thiols [99% of glutathione (GSH) is kept in erythrocytes, and about two-thirds of extracellular cysteine/cystine is in a bound form]. The Cys34 residue is able to neutralize such ROS and RNS as hydrogen peroxide (H_2_O_2_), peroxynitrite (ONOO^−^), superoxide anion, and hypochlorous acid (HOCl), being oxidized to sulfonic acid (HSA–SOH). Albumin ability to bind polyunsaturated fatty acids (PUFAs) and bilirubin can inhibit lipid peroxidation [2, 3].

Furthermore, albumin is easily modulated due to its flexible structure. The interaction of albumin with active species and oxidation of Cys34 can lead to an alteration of the protein binding properties toward the ligands, in particular pharmaceuticals and toxic substances. Additionally, the binding of some compounds affects the reactivity of the thiol group of Cys34 and modulates the antioxidant properties of the protein in the direction of strengthening or weakening. Undoubtedly, these properties of albumin should be taken into account in the development of therapy for pathologies associated with oxidative stress [2, 3].

*Moringa oleifera* is not called the Miracle Tree without reason. It possesses an interesting abundant antioxidant activity and acts as a good source of antioxidants due to the presence of several types of compounds, such as ascorbic acid, flavonoids, phenolics, and carotenoids. Antioxidant activity plays an important role in the protection against cell oxidation. The high content of phenolic compounds, such as flavonoids and phenolic acids, play an important role in the protection against cell oxidation. Furthermore, it is the main promotor of antibacterial, anti-inflammatory, and antitumor activity reported by many studies conducted on *M. Oleifera* leave extract. Some studies found cell apoptosis derived from cancer development can be cured with moringa because of its antioxidant potential, which scavenges the reactive oxygen radicals and, thus, avoids cell damage [4]. In *Curcuma xanthorrhiza*, the main component of antioxidants is curcuminoid, a group of phenolic compounds of curcumin, demethoxycurcumin, and bisdemethoxycurcumin. It can prevent lipid peroxidation and could serve as hepatoprotector [4, 5].

The albumin level of the patient increased from 2.1 gr/dL to 2.5 gr/dL after total 12 doses of supplement containing 7000 mg *C. striata*, 250 mg *M. oleifera*, and 250 mg *C. xanthorrhiza* extract along with the remarkable accelerating recovery of the patient. There are some possible explanations for this condition. First, as we know in critically ill patients, there is a burst of ROS production start from the early of pathological process. This patient suffered severe hemorrhagic shock due to prolonged placenta retention resulting in hypoxia and ischemia that worsened over time, especially in the last 24 hours before hospital admission. Resuscitation attempts also have a role in reperfusion injury, and it can aggravate ROS production. All these processes produce excessive ROS beyond the body’s neutralization capabilities. This high demand of antioxidant will use most of the antioxidants contained, including albumin, in *C. striata*, *M. oleifera*, and *C. xanthorrhiza* extract given to the patient, and will leave a small amount of albumin, which in turn will be used to increase albumin level in blood. This may cause the increase in albumin, which is not as aggressive as the recovery process [7, 8].

Second, prioritizing the need for antioxidants to neutralize the high level of ROS in the body, rather than increasing the amount of blood albumin, is a process in line with triage theory. The triage theory stated that some functions of micronutrients (40 essential vitamins, minerals, fatty acids, and amino acids) are restricted during shortage and that functions required for short-term survival take precedence over those that are less essential. Insidious changes accumulate as a consequence of restriction, which increases the risk of diseases of aging. Although there has been no further study regarding the implementation of this theory to antioxidants including albumin, it is well know that in critical condition, the body will prioritize vital organs to maintain life and sacrifice less vital organs [8, 9].

Accelerated recovery can be seen from length of stay (LOS). A study of 30,157 trauma-surviving patients from 164 trauma centers found that the average LOS in the ICU was 11.5 (median 7) days, and 26.3% of patients were female and 73.7% were male, with a mean age of 43 ± 20 years. Furthermore, this study describes the need for invasive ventilation will extended ICU stay on average by 3.1 days; transfusion, massive transfusion, and hematological disturbance prolonged ICU stay for 0.7 days, 3.3 days, and 1.2 days, respectively. Sepsis and kidney failure had a higher impact on the ICU LOS, with addition for 7.8 days and 8.1 days for each condition. This result showed longer LOS in critically ill patients in the ICU than in this patient (11.5 days versus 4 days), whereas this patient was also ventilated and received blood transfusions [10]. After 4 days in the ICU, patient was discharged from the hospital. On the fifth day, the patient came to the obstetrics and gynecology clinic for check-up, and based on clinical examination, there were no signs and symptoms of infection of the operation wound, no bleeding, and no pain with normal vital signs. The patient had no knowledge of balanced nutritious food and comes from a low income family, so she was educated to increase protein intake in her daily meals from affordable foods such as tofu and eggs. She was instructed to control after 2 weeks.

There were some limitations to this study. First, accelerated recovery based on clinical improvement and laboratory parameters relied on patient condition, blood gas analysis, blood routine examination, and renal and liver function. Albumin is one of indicators of antioxidant examined periodically in this patient, but there is no free radical parameter calculated. Second, this case report shows a very promising result. but a larger number of patients and a more specific laboratory examination is needed for valid results.

## Conclusion

This case report is very special because it has never been reported before where a patient with severe hemorrhagic shock due to prolonged placental retention was given oral *C. striata*, *M. oleifera*, and *C. xanthorrhiza* extract. This case report opens up the possibility of further research with larger number of patients to study the effect of oral *C. striata*, *M. oleifera*, and *C. xanthorrhiza* in reducing and neutralizing excessive free radicals and accelerating recovery of critically ill patients in the ICU.

## Data Availability

The ƒset and materials from this study are available from the corresponding author.
